# Supplementary material for: Uncertainties in critical slowing down indicators of observation-based fingerprints of the Atlantic Overturning Circulation
Source: Nat Commun. 2023 Dec 15;14:8344. doi: 10.1038/s41467-023-44046-9 (PMC10724135; doi:10.1038/s41467-023-44046-9)
Supplement: Supplementary file 1 — Supplementary Information [file 41467_2023_44046_MOESM1_ESM.pdf]

Uncertainties in critical slowing down indicators of observation-based fingerprints of the Atlantic Overturning Circulation - Supporting Information

Maya Ben-Yami[1,2*], Vanessa Skiba[2], Sebastian Bathiany[1,2], Niklas Boers[1,2,3]

[1*]Earth System Modelling, School of Engineering and Design, Technical University of Munich, Munich, Germany.
[2]Potsdam Institute for Climate Impact Research, Potsdam, Germany.
[3]Department of Mathematics and Global Systems Institute, University of Exeter,Exeter, UK.

*Corresponding author(s). E-mail(s): maya.ben-yami@tum.de;

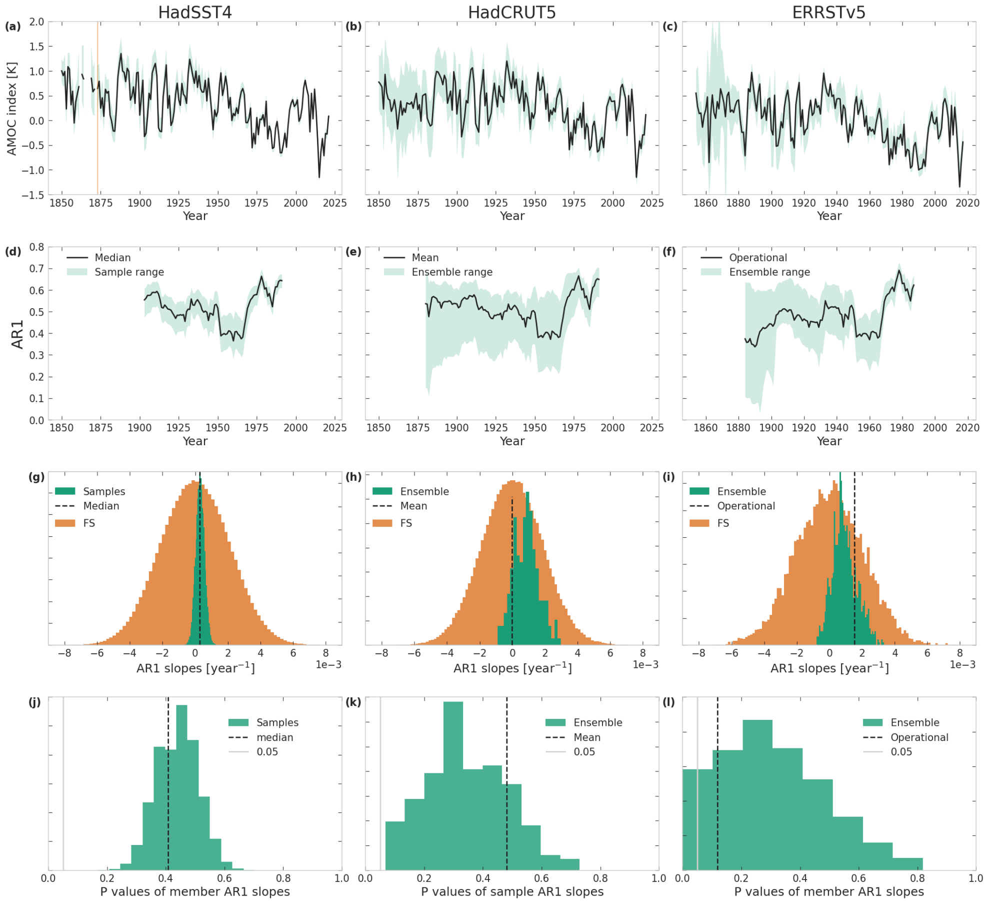

Fig. S1 Uncertainty of the autocorrelation (AR1) calculated from SST-based fingerprints. The increase in AR1 is not significant for either of the three datasets, although there is a large spread of p-values and for HadCRUT5 and ERSSTv5 some ensemble members do show a significant increase.

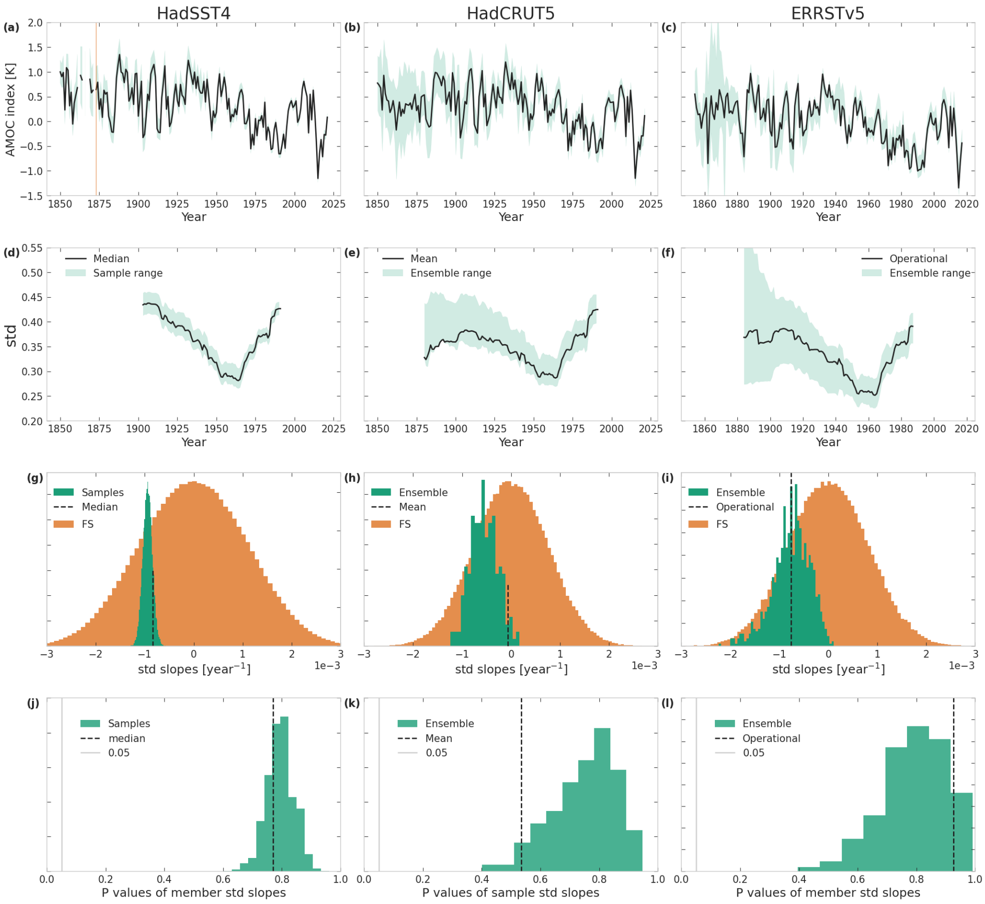

Fig. S2 Uncertainty of the standard deviation (STD) calculated from SST-based fingerprints. The STD for the AMOC index in HadSST4, HadCRUT5 and ERSSTv5 shows a marked difference from that of HadISST (see Fig S10 and B21, Fig. 3e). The overall negative trends of variance are because of a decrease in variance from 1900 to 1960, present in all three datasets shown here but not in HadISST. This is likely due to the changing number of observations in the subpolar gyre (see Fig. 1 in the main text): as the number of observations increase the index is a mean of more values and the variance decreases.

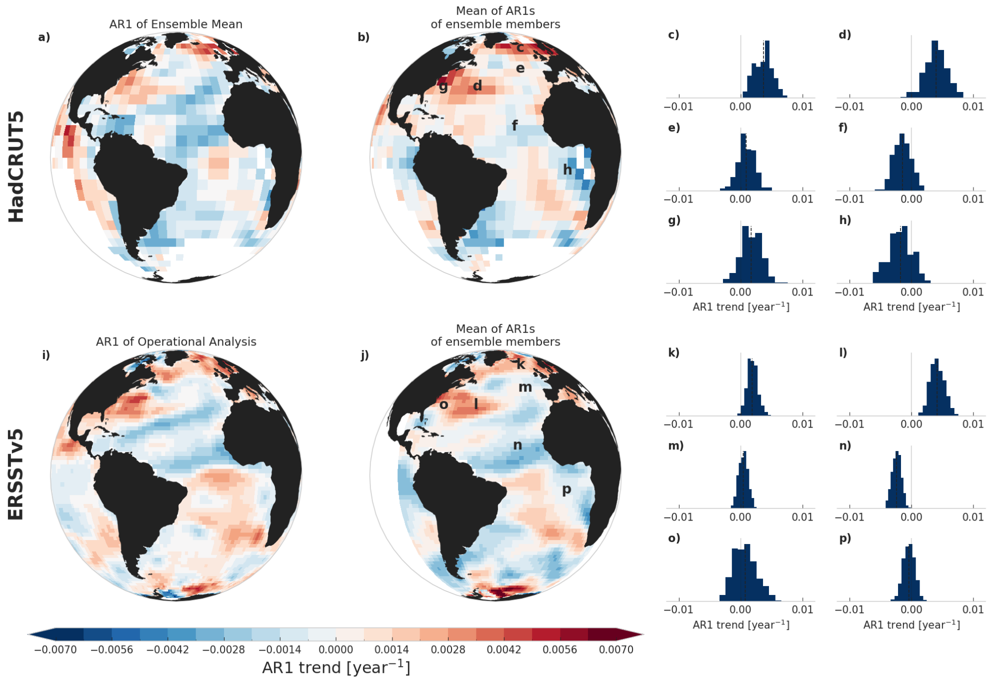

Fig. S3 Spatial representation of trends of the autocorrelation (AR1) calculated from the sea-surface temperature uncertainty ensembles.

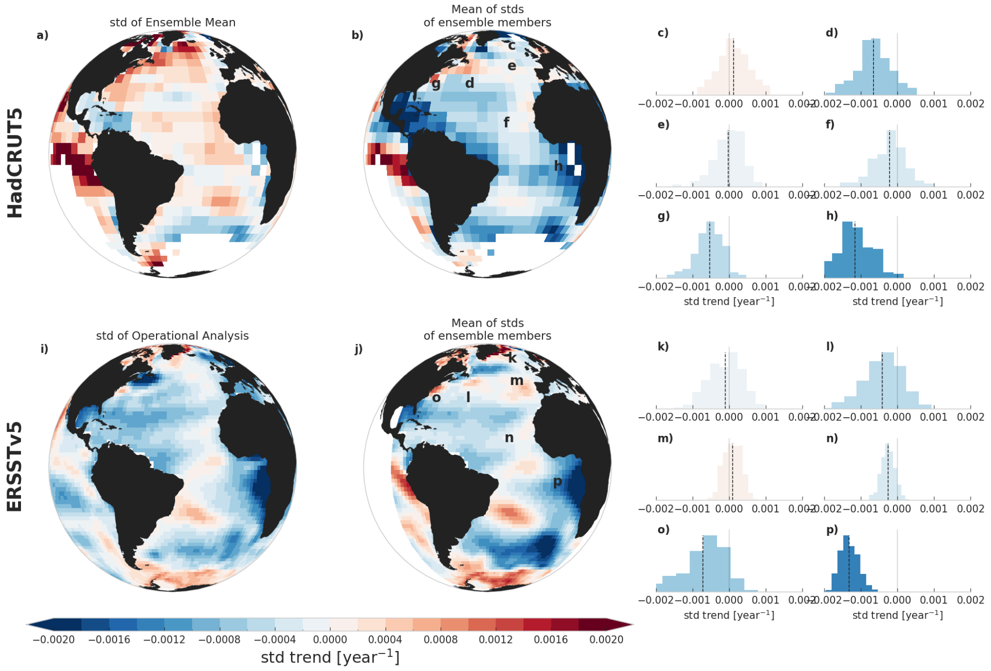

Fig. S4 Spatial representation of trends of the standard deviation (STD) calculated from the sea-surface temperature uncertainty ensembles. For HadCRUT5 the STD of the ensemble members is different from the STD of the ensemble mean because taking the mean removes the high variability at early times that causes the negative trend in the STDs seen in plots c-e (see also black line vs green range in Fig. S2d,f and h).

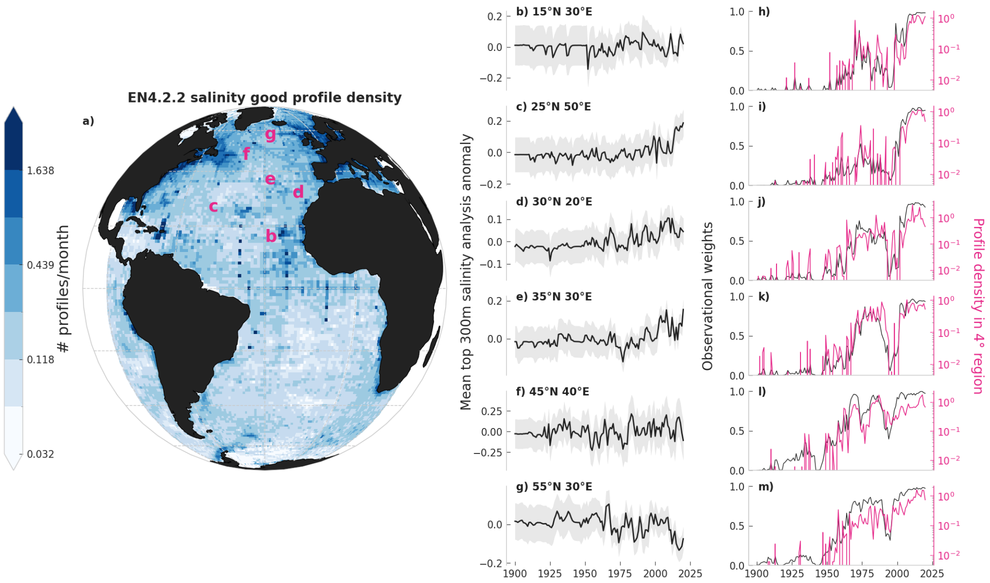

Fig. S5 Density of salinity profiles in the EN4.2.2 dataset for the Atlantic Ocean. a. Number of good salinity profiles per month in the Atlantic Ocean for the EN4.2.2 dataset (note the logarithmic scale). b-g. Mean annual salinity in the upper 300m (black) with its corresponding analysis uncertainty range (grey shading) for six locations in the North Atlantic. h-m. Mean annual observational uncertainty (black) and the density of good salinity profiles in the surrounding 4 region (pink) for the same six locations.

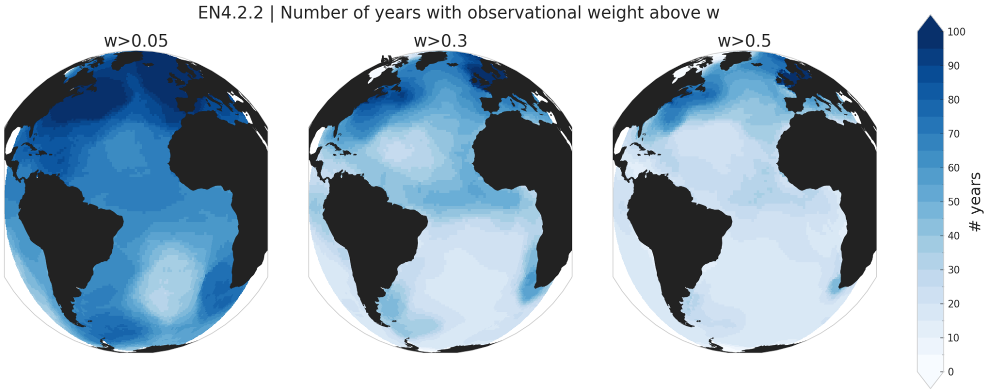

Fig. S6 Number of years in the EN4.2.2 data with specific observations weights. Top 300m mean observational weight above w = 0.05 (left), 0.3 (middle) and 0.5 (right). As in other figures only the region between 90°W and 30°E is shown.

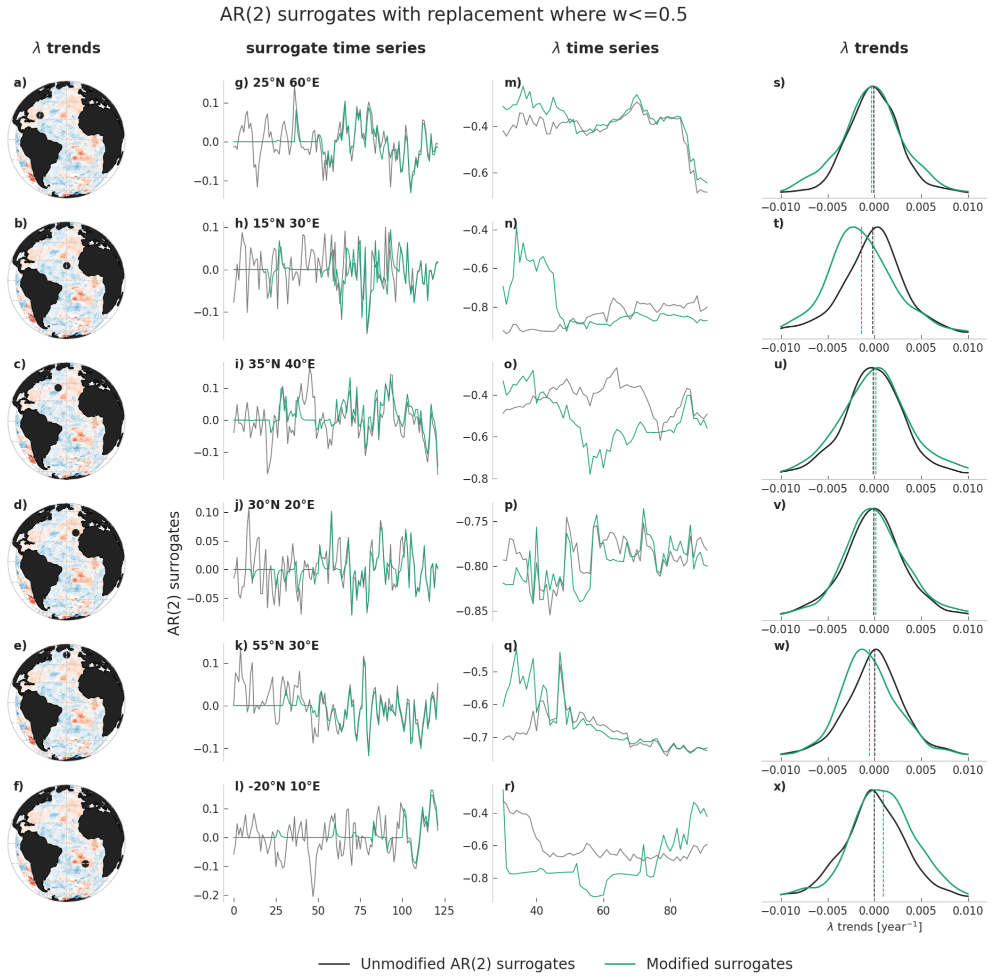

Fig. S7 Modification of AR2 surrogates of salinity time series. See Methods for details. First column: mean of distribution of modified surrogate λ trends. Second column: original AR2 surrogate (black) and modified AR2 surrogates where $w \leq 0.5$ (turquoise). Third column: λ time series of the time series shown in the second column. Fourth column: distribution of 1000 λ trends at the location, with a dashed line of the corresponding colour showing the mean. Note that the modification does not systematically bias the trends.

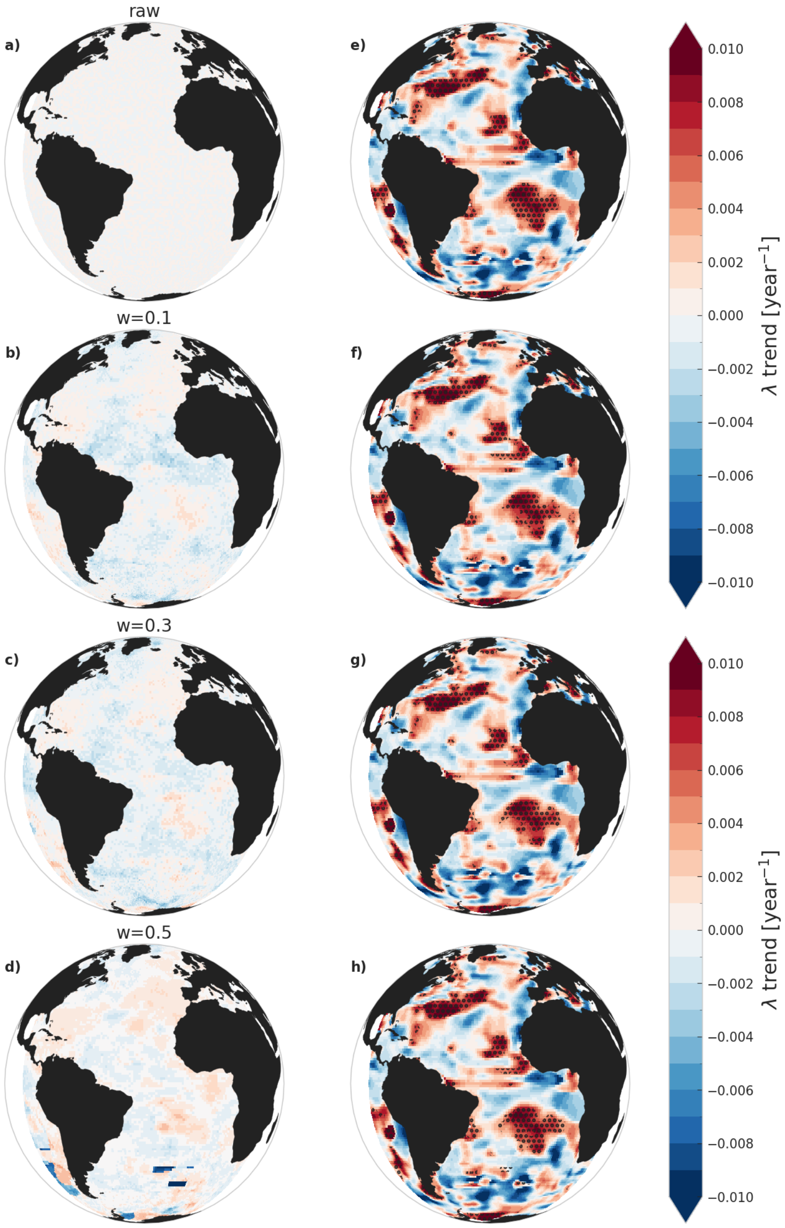

Fig. S8 Spatial distribution of surrogate trends. a. Mean of distribution of surrogate λ trends for raw AR2 surrogates of salinity time series, and modified surrogates with observational weight limit b. w = 0.1, c. 0.3 and d. 0.5. The areas where the analysis trends are significant given each of the surrogate distributions is shown in the second row on top of the λ trend of the analysis data (e-h). Note the differences in scale between the induced negative and positive biases (upper row) and the actually inferred trends (lower row). As in other figures only the region between 90°W and 30°E is shown.

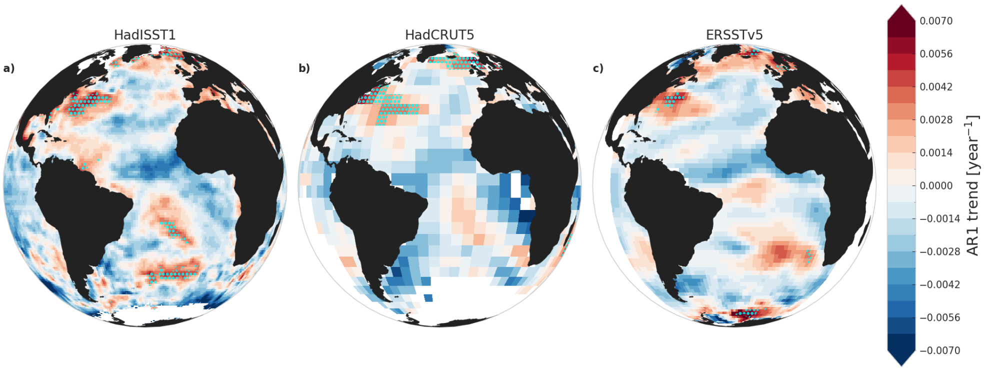

Fig. S9 Statistical significance of trends of the autocorrelation (AR1) calculated from the sea-surface temperature uncertainty ensembles.

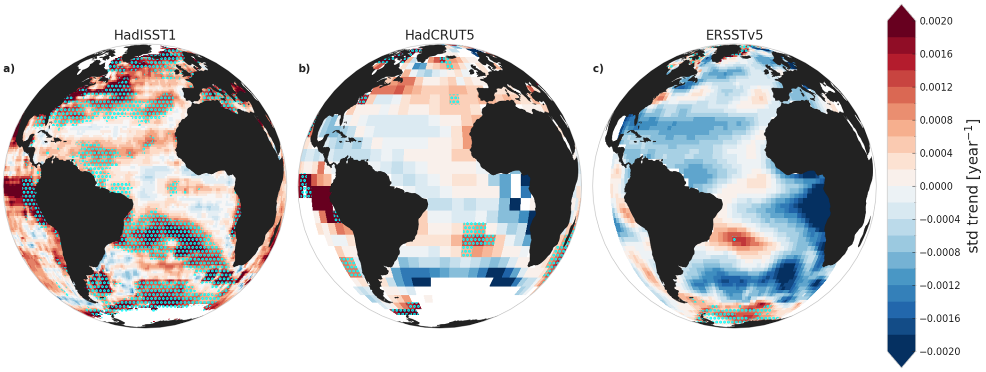

Fig. S10 Statistical significance of trends of the standard deviation (STD) calculated from the sea-surface temperature uncertainty ensembles. The differences between the trends for HadISST, HadCRUT5 and ERSSTv5 are likely due to the different interpolation methods employed in these datasets, which have a large influence on the variance.

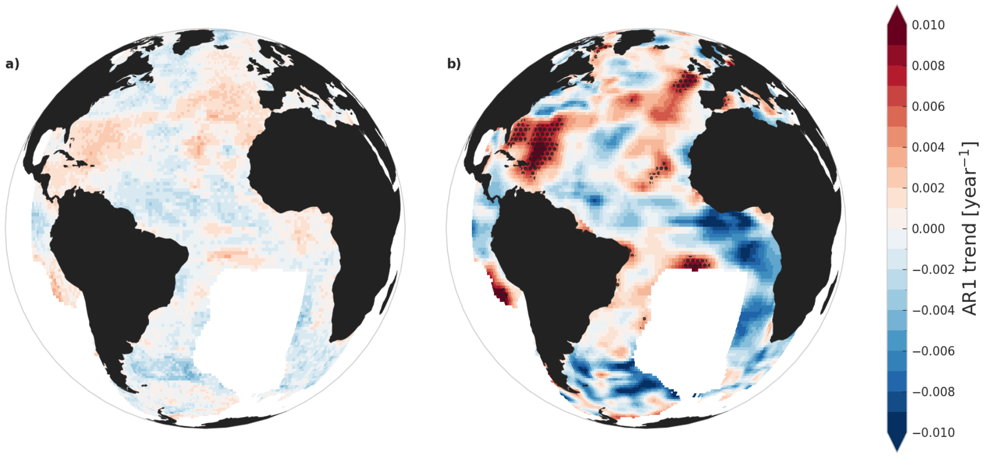

Fig. S11 Statistical significance of trends of the autocorrelation (AR1) calculated from the EN4.2.2 salinity dataset. The significant regions are shown for both, the raw AR(2) surrogates (a) and the modified surrogates with $w = 0.5$ (b). The general location of positive and negative trends is similar to that for λ, but the regions of significance are different. Note that in this case the significance regions are slightly reduced when using the modified surrogates, as the modification causes a larger false increase in AR(2) than it does in λ. Nevertheless, the overall pattern of regions with significant increases remains qualitatively the same.

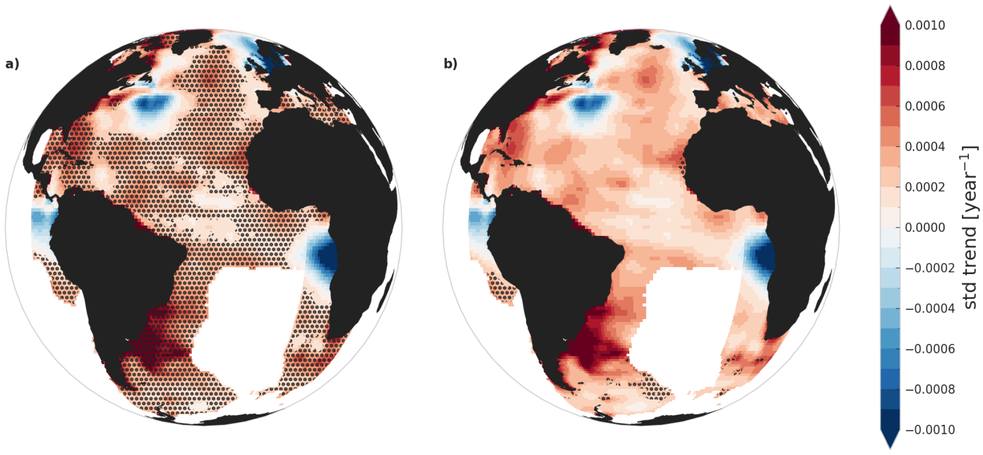

Fig. S12 Statistical significance of trends of the standard deviation (STD) calculated from the EN4.2.2 salinity dataset. Although almost the whole of the Atlantic shows an increasing variance, using modified surrogates removes all of the significant regions because the modification mimics the false variance increase that the EN4.2.2 analysis method causes. The areas of strong negative trend close to the North American and South African coasts are also due to dataset irregularities (a "spike" in the early 1900, probably due to a faulty data point). Note that these results do not rule out the existence of increasing variance trends in the true salinity values, as those would be masked by the analysis method that causes the false trends.

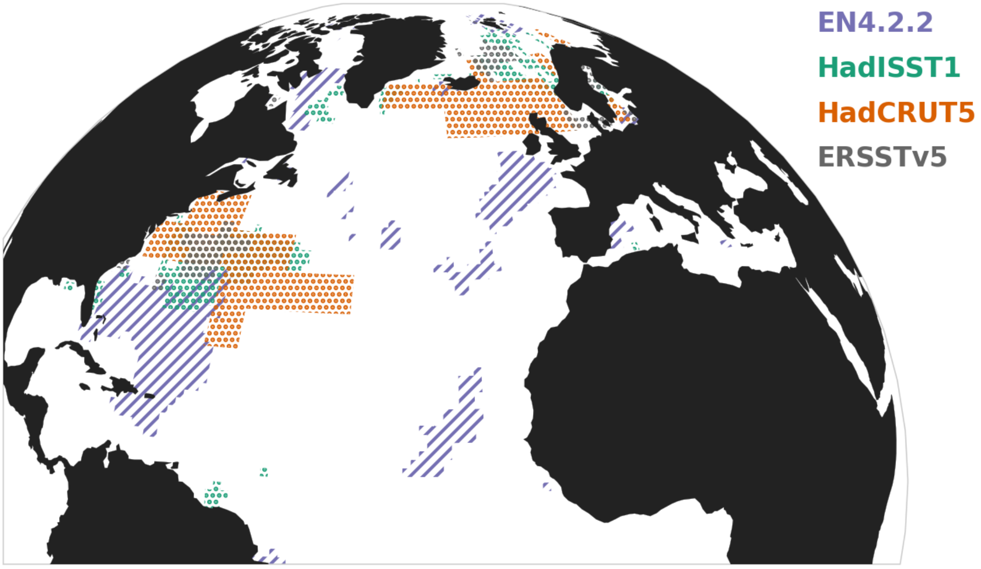

Fig. S13 Regions in the North Atlantic with a statistically significant increase in autocorrelation (AR1). Similarly to λ, the significance regions for the three datasets are roughly in the same area. The regions in the Irminger, Greenland and Iceland seas are similar to those found for λ. However the significant regions in lower latitudes are the northern Gulf Stream and its extension into the Atlantic Ocean.

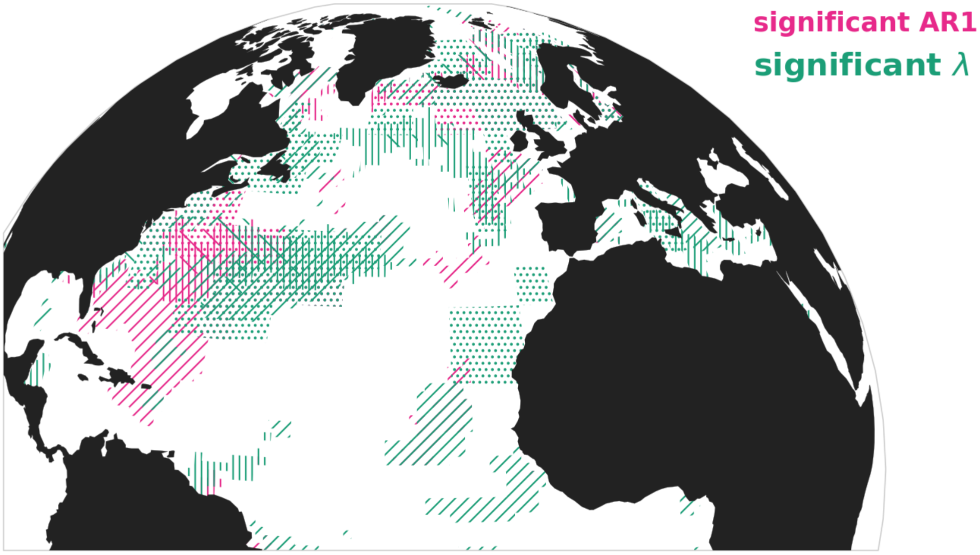

Fig. S14 Regions in the North Atlantic with a statistically significant increase in both the autocorrelation (AR1) and the corrected restoring rate λ. All the significant AR1 (λ) regions are shown in pink (turquoise). The three datasets are distinguished by pattern: dots, vertical lines upwards diagonal lines and downwards diagonal lines for HadCRUT5, HadISST, EN4.2.2 and ERSSTv5, respectively. The difference between the two indicators is due to the calculation method of λ, which accounts for autocorrelated residual noise. Together the regions for both indicators trace the currents that are part of the AMOC in the North Atlantic: from the subtropical gyre to the Gulf Stream along the North Atlantic Current and into the Greenland, Iceland, Irminger and Labrador seas.

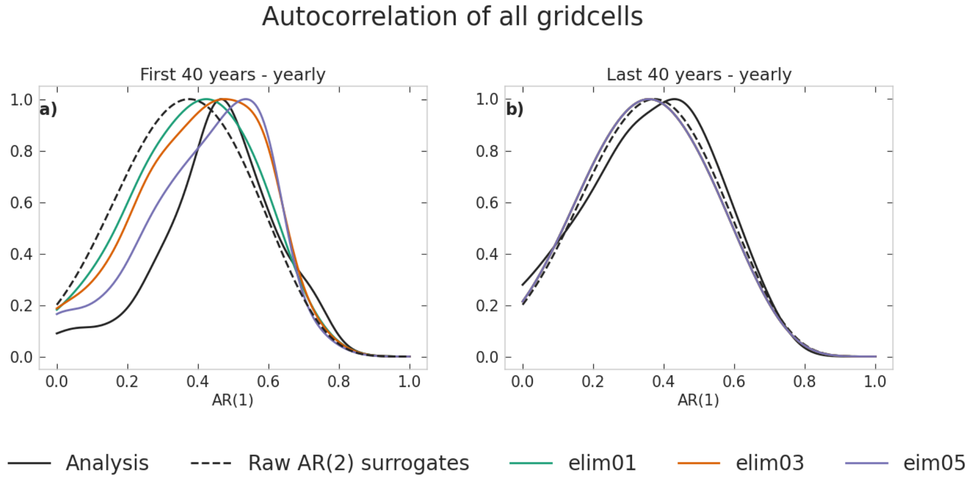

Fig. S15 Comparison of autocorrelation from all gridcells for the first 40 years and the last 40 years in the EN4.2.2 dataset. To show the difference between the first and last 40 years of salinity analysis data and the effect that modification has on the surrogates, we plot the distribution of the lag-one autocorrelation values of all Atlantic grid cells in the EN4.2.2 dataset in the first (a) and last (b) 40 years of data. The analysis autocorrelation (solid black) changes from a sharply peaked distribution in the first 40 years to a wider one in the last 40 years. The raw AR(2) surrogates (dashed black) match the last 40 years, but not the first 40. The modified surrogates with weight limits of 0.1 (turqouise), 0.3 (orange) and 0.5 (purple) have a more similar distribution to the analysis in the first 40 years, and are unchanged from the raw surrogates in the last 40 years, as required.
